# Raw Sap Consumption Habits and Its Association with Knowledge of Nipah Virus in Two Endemic Districts in Bangladesh

**DOI:** 10.1371/journal.pone.0142292

**Published:** 2015-11-09

**Authors:** Nazmun Nahar, Repon C. Paul, Rebeca Sultana, Emily S. Gurley, Fernando Garcia, Jaynal Abedin, Shariful Amin Sumon, Kajal Chandra Banik, Mohammad Asaduzzaman, Nadia Ali Rimi, Mahmudur Rahman, Stephen P. Luby

**Affiliations:** 1 ICDDRB, Dhaka, Bangladesh; 2 Swiss Tropical and Public Health Institute, Basel, Switzerland; 3 FHI360, Washington DC Office, Washington, D.C., United States of America; 4 Institute of Epidemiology, Disease Control and Research (IEDCR), Dhaka, Bangladesh; 5 Infectious Diseases and Geographic Medicine, Stanford University, Stanford, California, United States of America; NIH, UNITED STATES

## Abstract

Human Nipah virus (NiV) infection in Bangladesh is a fatal disease that can be transmitted from bats to humans who drink contaminated raw date palm sap collected overnight during the cold season. Our study aimed to understand date palm sap consumption habits of rural residents and factors associated with consumption. In November-December 2012 the field team interviewed adult respondents from randomly selected villages from Rajbari and Kushtia Districts in Bangladesh. We calculated the proportion of people who consumed raw sap and had heard about a disease from raw sap consumption. We assessed the factors associated with raw sap consumption by calculating prevalence ratios (PR) adjusted for village level clustering effects. Among the 1,777 respondents interviewed, half (50%) reported drinking raw sap during the previous sap collection season and 37% consumed raw sap at least once per month. Few respondents (5%) heard about NiV. Thirty-seven percent of respondents reported hearing about a disease transmitted through raw sap consumption, inclusive of a 10% who related it with milder illness like diarrhea, vomiting or indigestion rather than NiV. Respondents who harvested date palm trees in their household were more likely to drink sap than those who did not own date palm trees (79% vs. 65% PR 1.2, 95% CI 1.1–1.3, p<0.001). When sap was available, respondents who heard about a disease from raw sap consumption were just as likely to drink it as those who did not hear about a disease (69% vs. 67%, PR 1.0, 95% CI 0.9–1.1, p = 0.512). Respondents’ knowledge of NiV was low. They might not have properly understood the risk of NiV, and were likely to drink sap when it was available. Implementing strategies to increase awareness about the risks of NiV and protect sap from bats might reduce the risk of NiV transmission.

## Introduction

Nipah virus (NiV) infection is an emerging zoonosis that causes severe disease in both animals and humans. Several human Nipah virus outbreaks have been reported in Bangladesh since 2001 with a case fatality of 73% [1]. Multiple lines of evidence support a causal relationship between raw date palm sap consumption and human infection with Nipah virus. *Pteropus* bats, the reservoir of Nipah virus [2], occasionally shed Nipah virus in their saliva [3–5]. Infrared photography demonstrates *Pteropus* bats in Bangladesh frequently directly contaminate raw date palm sap with their saliva [6, 7]. Laboratory studies demonstrate that Nipah virus survives for several days in date palm sap [8]. Laboratory animals exposed to date palm sap spiked with Nipah virus develop Nipah infection [8]. Finally, several outbreak investigations in Bangladesh have found that people with Nipah virus infection are more likely to have drunk raw date palm sap than controls [7, 9–11]. Person-to-person transmission of human NiV has also been identified [12–14], a finding that has broader public health implications including the risk of a pandemic [15].

Raw date palm sap is harvested during the cold season, from November to March, by shaving the bark of the tree [16]. Often using a small wooden pipe, sap is collected into a clay pot and made available for people to drink raw [11, 16]. Bats frequently visit the harvested date palm trees and lick the sap stream that flows from the shaved part of the tree to the collection pot [6]. *Pteropus* bats can shed NiV through their saliva and urine [3–5] and can contaminate the date palm sap.

Since 2005, the messages “do not drink raw sap” or “avoid drinking raw sap” have been disseminated in outbreak affected communities in response to NiV outbreaks. Due to repeated NiV outbreaks associated with raw date palm sap consumption [7, 11], beginning in 2011, the Institute for Epidemiology, Disease Control and Research (IEDCR) of the Government of Bangladesh adopted an official strategy to discourage drinking raw date palm sap to prevent the transmission of NiV from bats to humans. The message was communicated via national newspapers and loudspeaker announcements in NiV affected areas.

We conducted an assessment to measure the existing raw sap consumption behavior and knowledge about NiV in two endemic districts. We intended this measurement as a baseline to help assess the effectiveness of a subsequent intervention. The objective of this analysis was to describe raw date palm sap consumption habits among rural community residents, grouping them by gender, district of residence, education and ownership of date palm trees, to determine if those factors and knowledge about NiV were associated with raw sap consumption.

## Methods

In this paper we presented baseline data of an intervention trial selecting Rajbari as an intervention district and Kushtia as a control district. From each district, we purposely selected two sub-districts using specific criteria. We chose rural sub-districts that did not border each other (between the intervention and control areas) to avoid spillover that could affect the study outcome. In Rajbari, we chose sub-districts with TV coverage via local satellite operators to be able to broadcast a public service announcement on close-circuit television. Finally, we looked for sub-districts where human population and date palm tree densities were similar. Although Rajbari and Kushtia districts experienced NiV outbreaks in the past, after our selection we found that our selected sub-districts had not experienced any recognized outbreak.

Based on the sample size calculation to evaluate the impact of an intervention, we randomly selected 75 villages per district (two sub-districts from each district) from the list of villages from the 2011 census [17] for a cross sectional survey. We planned to enroll 450 respondents per gender per district, for a total of 1,800 respondents altogether, allowing for a 15% refusal/absent rate. In each village, we interviewed six men and six women to understand their date palm sap consumption habits and their knowledge about NiV transmission.

The data collection team conducted the survey between 21 November and 7 December 2012. Among men, the team interviewed the main income earner of a household. Among women, the team interviewed the wife of the main male income earner in the household. To select the first man respondent randomly, the team identified the closest household to where the most recent birth in the village occurred. Similarly, to select the first woman respondent randomly, the team identified the closest household to where the most recent wedding occurred. To conduct the interviews, the field team approached the selected household. If the desired respondent was available and provided informed consent, they interviewed the person. They conducted face-to-face-interviews with men and women using two separate pre-tested standardized questionnaires. If the person was not available, they revisited the household twice within the next 24 hours. After that time-period, if not available, the respondent was skipped and not replaced.

After interviewing a respondent, the field team skipped the next closest 20 households and then approached the 21st for consent and enrolment of a respondent. They repeated this procedure until the required number of households per gender per village had participated in the survey. People were excluded if someone else in the household had already been enrolled.

The team asked both men and women about their knowledge about NiV infection and their own raw sap consumption. Since women spend more time at home, where sap consumption usually occurs, the team only asked women about the amount of raw sap consumed per-week by household members. To collect the information on the amount of raw sap consumed, the team asked the women respondents how many people in the household drank raw sap, how many days a week and how many glasses of raw sap they usually consumed during the previous sap collection season.

### Data analysis

We calculated the proportion of men and women and all household members drinking raw sap during the last sap collection season, the frequency and amount consumed per capita and source of raw sap consumed. We calculated the amount of raw sap consumed assuming that each glass of sap was 250 ml. We compared sap consumption practices by gender and study area/district using a t-test, adjusted for cluster effects [18]. To investigate if there was an association between raw sap consumption and gender, district, education, ownership of date palm trees and having heard of NiV, we restricted the analysis to the availability of raw sap. Thus, we excluded people who reported not drinking raw sap because of unavailability. We calculated prevalence risk ratios (PR) using a log linear model and 95% confidence interval (CI) adjusted for village level clustering.

### Ethical consideration

The team obtained written informed consent prior to conducting the survey. Ethical Review Committee of icddr,b and Office of International Research Ethics of FHI 360 reviewed and approved the protocol.

## Results

### Background information of respondents

During the survey, the field team enrolled 1,777 (99%) of the 1,800 targeted participants, equally distributed among districts and by gender. The mean age of the respondents was 40 years. Men respondents were older than women respondents (mean 45 vs. 34 years). Less than half (42%) completed primary school education. More respondents in Kushtia District completed primary education compared to Rajbari District. More women respondents in both districts completed primary education than men respondents (Table 1).

**Table 1:** Demographic characteristics, knowledge about Nipah and raw sap consumption habits in the previous sap collection season by gender and district.

Characteristics	Gender^§^		District^§§^		
	Men (N = 889)	Women (N = 888)	Rajbari (N = 892)	Kushtia (N = 885)	Total (N = 1,777)
	n (%)	n (%)	n (%)	n (%)	n (%)
Mean Age (Standard Deviation)	45 (14.2)	34 (9.8) ^†††^	40 (13.8)	39 (13.1)	40 (13.4)
Completed primary education	338 (38)	413 (47) ^†††^	341 (38)	410 (46) ^†††^	751 (42)
**Knowledge about NiV**					
Heard about a disease that can be transmitted from bats to people	159 (18)	150 (17)	176 (20)	133 (15) ^†^	309 (17)
Heard about a disease from raw sap consumption	320 (36)	341 (38)	322 (36)	339 (38)	661 (37)
Heard the name “Nipah” disease	29 (3)	60 (7) ^†††^	47 (5)	42 (5)	89 (5)
**Individual level consumption**					
Ever drank raw sap	851 (96)	829 (93)	857 (96)	823 (93)	1,680 (95%)
Drank raw sap last season	495 (56)	396 (45) ^†††^	382 (43)	509 (57) ^†††^	886 (50)
**Raw sap drinking frequency during last season**					
At least once or twice a month	281 (32)	383 (43) ^†††^	284 (32)	380 (43)	664 (37)
Once or twice a season	214 (24)	8 (1) ^†††^	96 (11)	126 (14)	222 (12)
**Source of raw sap drank during the last season**					
Purchased from neighboring *gachhi* or tree owner	199 (22)	148 (16)	159 (18)	188 (21)	347 (20)
Own household trees	105 (12)	81 (9)	99 (11)	87 (10) ^††^	186 (10)
Gift	81 (9)	112 (13) ^†††^	91 (10)	102 (11)	193 (11)
Market	97 (11)	29 (3) ^†††^	43 (5)	83 (10) ^†^	126 (7)
Mobile vendor	75 (8)	56 (6)	22(2) ^†††^	109(12) ^†††^	131 (7)
**Household level raw sap consumption**					
A least one person in the household consume raw sap in the last season*		472 (53)	194 (44)	278 (63)	472 (53%)
Mean number of household members (95% confidence interval)*		4.5 (4.43, 4.69)	4.8 (4.61, 5.02)	4.3 (4.15, 4.47)	4.5 (4.43, 4.69)
Mean number of household members who drank raw sap in the sap drinking household (95% confidence interval) *		3.5 (3.31, 3.62)	3.5 (3.21, 3.73)	3.5 (3.26, 3.66)	3.5 (3.31, 3.62)
**Per capita raw sap consumption at household per week in the peak month during the last season (in liters)** *					
Mean (95% confidence interval) and [range]		0.6 (0.55,0.62) [0.14, 5.3]	0.6 (0.55, 0.67) [0.14, 5.3]	0.6 (0.52, 0.62) [0.14, 2.6]	0.6(0.55,0.62) [0.14, 5.3]
**Use of any raw sap at household level in the last season**					
Drank raw sap	546 (61)	481 (54) ^†^	457 (51)	570 (64) ^†††^	1,027 (58)
Made molasses	94 (11)	77 (9)	112 (13)	59 (7)	171 (10)
Shared with neighbors and relatives	74 (8)	70 (8)	73 (8)	71 (8)	144 (8)
Sold raw sap	15 (2)	6 (1)	11 (1)	10 (1)	21 (1%)
Feed sap to animals	10 (1)	6 (1)	9 (1)	7 (1)	16 (1)
Made *tari*	0 (0)	1 (0)	0 (0)	1 (0)	1 (0)

* Calculated from the response of women respondents

^§^ P value was calculated by comparing men and women (by gender)

^§§^ P value was calculated by comparing Rajbari and Kushtia Distirct (by district)

^†^P value < 0.05

^††^P value < 0.01

^†††^P value< 0.001

P-values were cluster adjusted

### Knowledge about NiV

More than one third (37%) of respondents reported hearing about a disease that resulted from raw sap consumption, 17% of respondents heard about a disease transmitted from bats to people and 5% of respondents had heard of a disease named “Nipah” (Table 1). Respondents who mentioned about a disease resulting from raw sap consumption related it with a number of illnesses like diarrhea and vomiting (10%), *gastric* (1%) and other health problems in addition to a deadly disease (12%) and NiV (2%).

More respondents from Rajbari (20%) mentioned hearing about a disease that can be transmitted from bats to people than respondents from Kushtia (15%); more women (7%) reported hearing about a disease named “Nipah” than men (3%) (Table 1).

### Individual habits of raw sap consumption

Half of the respondents (50%) reported drinking raw sap during the previous sap collection season, 37% of respondents consumed raw sap at least once per month. Twenty percent of all respondents purchased raw sap from *gachhis* in their neighborhood. More men drank raw sap than women (56% vs. 45%) and more respondents from Kushita District (57%) drank raw sap than respondents from Rajbari District (43%) during the previous sap collection season (Table 1).

### Household use of raw date palm sap

Respondents reported that their household members primarily used raw date palm sap for consumption, followed by making molasses and sharing raw sap with neighbors and relatives (Table 1). On average, households consisted of 4.5 members and 3.5 members drank raw sap in the previous sap collection season. For households reporting any consumption in the previous sap collection season, per capita mean raw sap consumption averaged about half a liter per week during the peak month of sap collection, i.e. mid December to mid February.

### Reason for not drinking raw sap

The primary reason that respondents gave for not drinking raw sap during the previous sap collection season was because the sap was unavailable or they did not enjoy drinking it (Table 2). Only 6% of respondents mentioned that they did not drink sap because they heard about “Nipah” or heard about a disease from drinking raw sap that caused death.

**Table 2:** Respondents reported caused for not drinking raw date palm sap during previous sap collection season from Rajbari and Kushtia Districts, 2012.

Causes for not drinking raw sap during the last sap collection season* †	Total (N = 794)
	n (%)
Sap was not available	470 (59)
Did not like to drink	158 (20)
Did not purchase	51 (6)
Risk of disease	47 (6)
Heard about “Nipah” or heard death after drinking raw sap	45 (6)
Too expensive	33 (4)

* Number of people who did not consume raw sap in the previous season was used as the denominator

^†^ Open ended responses with multiple responses

### Associations with raw sap consumption

When raw sap was available men were somewhat more likely than women to drink it (74% vs. 62% PR 1.1, 95% CI 1.1–1.2, p<0.001) (Table 3). Kushtia District residents were somewhat more likely than Rajbari District residents to drink raw sap (72% vs. 62% PR 1.1, 95% CI 1.1–1.2, p = 0.002). Respondents whose households owned date palm trees harvested during the previous sap collection season drank sap more frequently than those respondents whose household did not own trees drank (79% vs. 65% PR 1.2, 95% CI 1.1–1.3, p<0.001). Neither respondents' education nor their knowledge about NiV was associated with raw sap consumption (Table 3).

**Table 3:** Characteristics associated with raw sap consumption among those living in villages that had access to date palm sap during previous sap collection season from Rajbari and Kushtia Districts, 2012.

Characteristic	Consumption of raw sap %	Prevalence ratio (95% Cl)	*P value* *
**Gender**			
Women	*62 (391/635)*		
Men	*74 (494/672)*	*1*.*1 (1*.*1–1*.*2)*	*<0*.*001*
**District**			
Rajbari	*62 (379/608)*		
Kushtia	*72 (506/699)*	*1*.*1 (1*.*0–1*.*2)*	*0*.*002*
**Education**			
<5 years	*68 (500/732)*		
5 to 9 years	*66 (276/418)*	*0*.*9 (0*.*8–1*.*0)*	*0*.*441*
10 to 12 years	*69 (109/157)*	*0*.*9 (0*.*8–1*.*1)*	*0*.*799*
**Household own date palm trees**			
No	*65 (705/1,079)*		
Yes	*79 (180/228)*	*1*.*2 (1*.*1–1*.*3)*	*<0*.*001*
**Heard about NiV transmission**			
Heard about a disease that can be transmitted from bats to people			
No	*68 (730/1,066)*		
Yes	*64 (155/241)*	*0*.*9 (0*.*8–1*.*0)*	*0*.*303*
Heard about a disease from raw sap consumption			
No	*67 (531/792)*		
Yes	*69 (353/514)*	*1*.*0 (0*.*9–1*.*1)*	*0*.*512*
Heard of “Nipah” disease			
No	*68 (836/1,230)*		
Yes	*64 (49/77)*	*0*.*9 (0*.*7–1*.*1)*	*0*.*482*

* Cluster adjusted

## Discussion

Rural Bangladeshi residents like drinking raw date palm sap. In our study, half of the respondents reported drinking it during the last sap collection season putting them at risk of contracting NiV. Those who did not drink raw sap, attributed it mostly to its unavailability. Since NiV kills most of the people infected [1], outbreaks often receive media attention [19–21]. The Government of Bangladesh has taken efforts to communicate the risk associated with raw date palm sap consumption through newspaper announcements as well as in the outbreak-affected communities using interpersonal and loudspeaker communication. However, respondents’ understanding of NiV is still low and only a small proportion of people had heard about NiV and its consequences. Reducing consumption of raw date palm sap would reduce the risk of NiV transmission. Concentrating on raising awareness about the disease and its associated risk, as well as disseminating information on a regular basis for several years may help increase the likelihood of long-term change [22].

Men in our study were somewhat more likely to drink raw sap than women. In rural Bangladesh men have higher mobility in the locality and more access to money than women [23]. As a result, they might have more options to drink sap. Similarly, respondents who had harvested trees at home were more likely to consume sap, presumably because it was easily available to them, at minimal or no cost. The result is similar to other food-focused studies that suggest that people consume more unhealthy food when it is readily available [24–26].

In our study, when sap was available, knowledge about the potential risk of NiV in this population did not influence raw sap consumption behavior, indicating a gap between knowledge and NiV risk perception. This behavior can be compared with raw milk consumption in the United States. Although many states restrict the sale of unpasteurized or raw milk to prevent foodborne disease outbreaks, some people still consume raw milk [27–29]. In the Portland, Oregon area, during an outbreak of *Escherichia coli* O157: H7 infection caused by raw milk consumption, messages were widely disseminated about the life threatening risk of raw milk consumption, however, sale of the raw milk continued until the dairy selling that milk was forced out of the retail business [30]. Milk consumers were skeptical about the inherent hazard [30]. A study among farm families in Pennsylvania suggests that farmers consume raw milk primarily because of the taste and availability (convenience) and because it is a traditional practice, less expensive than retail pasteurized milk [31].

NiV is a relatively newly identified disease and few people in our study sites have heard enough about it to understand the risk it represents. Those who heard about it might not be fully aware of NiV infection consequences, since many of them only related raw sap consumption with gastrointestinal distress like diarrhea and vomiting. They may not be concerned because they have been drinking sap for many years, and not experiencing any serious consequences, thus they may not consider themselves at risk. When people are concerned about their health, they consider themselves at risk and intend to change and make efforts towards change [32]. NiV is a disease with low probability of occurrence but with a high fatality rate, similar to other common exposure that occasionally result in human fatality. Lightning strikes, for example, affects people engaged in common outdoor activities. Although it is a well characterized risk, and recommended practices could reduce risks, many people ignore expert advice and consequently a small proportion are hit and killed by lightning each year [33]. Similar to the behavior of people who drink raw date palm sap, people who put themselves at risk of lightning strike, may not properly understand the risk, or may not consider themselves at risk since lightning does not strike people frequently.

A number of factors influence consumption of foods that have well understood adverse health effects [25, 26, 34–37]. For example, people consume sugar-sweetened beverages that contribute to obesity because those are easily available and inexpensive, advertised or promoted [34–36]. Behavioral intention consistent with negative and positive evaluation towards performing a behavior, perceived behavior control, and subjective norms based on how others approve or disapprove the behavior can influence peoples’ choice of drinking sweetened beverages [36]. Though some of them know the health consequences, they do not perceive themselves as vulnerable to weight gain and they see others drinking it [35]. Similarly, drinking raw date palm sap is widely acceptable and people see others drinking it. Even if they know about the risk, it is a seasonal delicacy craved during the sap-collection season and they may not perceive themselves as potential victims of NiV. Thus, it might be difficult to change people's behavior so that they decide to stop drinking raw sap altogether.

A potentially useful strategy would be to create awareness of NiV and to provide an option of drinking safer sap. Similar to substituting high-sugar soda with diet soda, an approach that allows people to drink sap while avoiding the risk of infection may be effective at reducing exposure to NiV. To keep sap clean during collection, local sap harvesters occasionally use a skirt-like barrier called *bana*, made from locally available materials, that covers the sap flow and the collection pot to interrupt bats' access [16]. The method was effective in protecting sap from bats [38]. Pilot studies to promote them found that *banas* were well accepted by sap harvesters. Many of them made and used *banas* on date palm trees used for raw sap consumption [39, 40]. In additon to raising NiV awareness, promoting *banas* might help people make a healthy decision on the risk of consuming raw date palm sap, understanding that there is a way to reduce risk without having to avoid consuming it.

In addition to reducing NiV transmission, the use of *banas* on trees for raw sap consumption could help reduce the risks of other diseases that could be transmitted through bats. Bats are the reservoir of several zoonoses that can affect both humans and other animals [41, 42]. In our study, some respondents reported vomiting and diarrhea after drinking raw sap. This suggests that bats might contaminate sap with other pathogens. For example, fruit bats in Bangladesh carry *Salmonella* [43] and presumably other enteropathogens.

Our study has limitations. From this survey we received only some reports of fermented sap preparation and consumption, though it is one of the routes of NiV transmission to humans [44, 45]. Drinking alcohol is proscribed in Islam and therefore it is a sensitive issue to report. Thus, from our study we cannot predict how many people consume, or how frequently they consume, fermented sap. Understanding more about practices related to sap fermentation and consumption could guide us to incorporate this issue in future intervention messages.

We do not fully understand why some people drank raw sap after knowing the risk of getting NiV or why residents of Kushtia were more likely to drink sap than residents of Rajbari. These would be useful topics for further exploration.

NiV is a serious disease that often kills people infected [1] and because it can be transmitted from person to person [12–14] there is some risk that, during human infection, the virus could evolve to become more easily transmissible from person-to-person, increasing the risk of a pandemic [46]. The large and increasing population density in Bangladesh means more human interactions, more mobility and therefore increased opportunities for person-to-person transmission that would further increase this global risk. Reducing the risk of NiV spillover by developing effective and practical interventions is in the interest of the global community. Such interventions will require more than simply informing people on the risk of drinking raw date palm sap, but even just informing them would be a sound first step.

## Supporting Information

S1 Dataset Dataset (DTA). (DTA)
